# Optimal Dose of Cefoperazone-Sulbactam for Acute Bacterial Infection in Patients with Chronic Kidney Disease

**DOI:** 10.3390/antibiotics11050610

**Published:** 2022-04-30

**Authors:** Chien-Ming Chao, Chih-Cheng Lai, Chen-Hsiang Lee, Hung-Jen Tang

**Affiliations:** 1 Department of Intensive Care Medicine, Chi Mei Medical Center, Liouying, Tainan 73657, Taiwan; ccm870958@yahoo.com.tw; 2 Department of Internal Medicine, Kaohsiung Veterans General Hospital, Tainan Branch, Tainan 710, Taiwan; dtmed141@gmail.com; 3 Division of Infectious Diseases, Department of Internal Medicine, Kaohsiung Chang Gung Memorial Hospital, Kaohsiung 833, Taiwan; lee900@cgmh.org.tw; 4 College of Medicine, Chang Gung University, Kaohsiung 833, Taiwan; 5 Department of Medicine, Chi Mei Medical Center, Tainan 71004, Taiwan; 6 Department of Medical Research, Chi Mei Medical Center, Tainan 71004, Taiwan

**Keywords:** cefoperazone-sulbactam, chronic kidney disease, dose

## Abstract

The optimal dosage of cefoperazone-sulbactam for patients with chronic kidney disease (CKD) remains unclear. This study aimed to investigate two treatment strategies of cefoperazone-sulbactam–2 g/2 g twice daily and adjusted dose according to renal function for patients with CKD. A total of 155 patients with CKD received cefoperazone-sulbactam either at a dose of 2 g/2 g twice daily (study group) or adjusted according to renal function (control group) for the treatment of acute bacterial infection. The primary outcome was the clinical response rate at day 14 and the secondary outcomes included treatment failure and all-cause death. The study group had a higher clinical response rate (80.0% vs. 65.0%) and a lower treatment failure rate (4.0% vs. 23.8%) as compared with the control group. Further multivariable analysis showed that compared with the control group, the study group had a higher clinical response rate (adjusted OR = 4.02; 95% CI, 1.49–10.81) and lower treatment failure rate (adjusted OR = 0.06; 95% CI, 0.01–0.28). In addition, no significant difference in all-cause mortality was observed between the study and the control group (adjusted OR = 1.95; 95% CI, 0.57–6.66). Finally, no significant difference was observed between the study and the control group in the risk of the adverse events (AEs)–diarrhea (*p* = 0.326), eosinophilia (*p* = 1.000), prolonged PT (*p* = 0.674), alteration in renal function (*p* = 0.938) and leukopenia (*n* = 0.938). In conclusion, cefoperazone-sulbactam at a dose of 2 g/2 g twice daily could achieve better clinical efficacy than the reduced dosage regimen. Additionally, this dosage did not increase the risk of AE compared to the reduced dose. Therefore, cefoperazone-sulbactam at a dose of 2 g/2 g twice daily is an effective and safe regimen for acute bacterial infection in patients with CKD.

## 1. Introduction

Cefoperazone-sulbactam is a broad-spectrum antibiotic that exhibits potent in vitro activity against the commonly encountered pathogens, including Gram-positive and Gram-negative organisms and anaerobes [1,2,3,4]. Moreover, the combination of sulbactam with cefoperazone largely enhanced their activity against multidrug-resistant organisms (MDROs), such as extended-spectrum β-lactamase-producing *Escherichia coli* and *Klebsiella pneumoniae*, and carbapenem-resistant *Acinetobacter baumannii* [5,6,7,8,9]. Furthermore, the clinical effectiveness of cefoperazone-sulbactam has been demonstrated in many types of infections, and cefoperazone-sulbactam has been indicated for infections of the respiratory tract, urinary tract, intra-abdominal, pelvic inflammatory disease, skin and soft tissue, and surgical site [10,11,12,13,14,15].

However, the optimal dose of cefoperazone-sulbactam for the patients with renal insufficiency remains unclear. Although the dose of cefoperazone needs to be adjusted in patients with both liver dysfunction and significant renal impairment, it does not need to be adjusted in patients with chronic kidney disease (CKD). In contrast, the dose of sulbactam needs to be adjusted according to the patient’s renal function. Additionally, a high dose of up to 6–9 g/day of sulbactam is needed to achieve a better response in the treatment of infection caused by MDROs, such as carbapenem-resistant *A. baumannii* [5,16,17]. Thus, if clinicians prescribe cefoperazone-sulbactam based on the dose adjustment of sulbactam according to the patient’s renal function, the dose of cefoperazone would be inadequate and the dose of sulbactam might be insufficient for treating MDROs. Hence, this study was conducted to assess the clinical efficacy and safety of cefoperazone-sulbactam at two different doses (2 g/2 g twice daily vs. an adjusted dosage according to the patient’s renal function) for the treatment of acute bacterial infection in patients with CKD.

## 2. Results

### 2.1. Demographic Feature of the Study Populations

A total of 155 patients with CKD receiving cefoperazone-sulbactam for the treatment of acute bacterial infection were included in this study (Table 1). Their mean age was 77.2 years (± 12.2) and 89 (57.4%) of the 155 patients were males. Lower respiratory tract infections were the most common type of infection (41.9%, *n* = 65), followed by urinary tract infections (35.5%, *n* = 55), primary bacteremia (9.0%, *n* = 14), skin and soft tissue infection (7.7%, *n* = 12) and intra-abdominal infection (2.6%, *n* = 4). Diabetes mellitus was the most common comorbidity (51.0%, *n* = 79), followed by malignancy (26.5%, *n* = 41). The mean Charlson comorbidity index and sequential organ failure assessment (SOFA) scores were 3.5 (± 2.2) and 4.3 (± 2.2), respectively. The mean duration of cefoperazone-sulbactam treatment was 7.0 days.

Compared to the control group with adjusted dosage according to renal function, the study group had more male patients, lower body weight, and urinary tract infections, and lower respiratory tract infections, diabetes mellitus, heart failure, renal function, Charlson score, and SOFA scores (all *p* < 0.05). Additionally, the study group had a shorter antibiotic duration than the control group (6.0 ± 2.4 vs. 7.9 ± 4.4, *p* < 0.001).

### 2.2. Clinical Outcomes

On day 14, the overall clinical response and treatment failure rates were 72.3% (*n* = 112) and 14.2% (*n* = 14.2%), respectively. The all-cause mortality rate was 13.6% (*n* = 21). The study group had higher clinical response and lower treatment rates (*p* = 0.002). Further multivariable analysis showed that the study group had higher clinical response (adjusted odds ratio [OR] = 4.02; 95% confidence interval [CI], 1.49–10.81) and lower treatment failure rates (adjusted OR = 0.06; 95% CI, 0.01–0.28) (Table 2). No significant difference in all-cause mortality rate was observed between the study and the control groups (adjusted OR = 1.27; 95% CI, 0.35–4.64).

### 2.3. Subgroup Analysis

First, a higher clinical response rate observed in the study group than in the control group was consistent in all the subgroup analyses. However, most of these differences were not statistically significant, except for the subgroup analysis of patients with other infections (OR = 13.7; 95% CI, 1.47–123.7). Second, compared to the control group, the study group had a lower treatment failure rate in all subgroup analyses. These differences remained significant in the subgroup analysis of males, females, SOFA score ≥ 4, Charlson comorbidity score > 3, CKD, stage 4, no dialysis, and other infections. Finally, these subgroup analyses did not reveal a significant difference in mortality rate between the study and control groups (Table 3).

### 2.4. Microbiological Investigations

In this study, *E. coli* was the most common causative pathogen (*n* = 49), followed by *K. pneumoniae* (*n* = 23), *Pseudomonas aeruginosa* (*n* = 19) and *A. baumannii* (*n* = 17). Among these pathogens, carbapenem resistance was noted in four *E. coli*, five *K. pneumoniae*, two *P. aeruginosa*, and two *Enterobacter cloacae* strains. The clinical response rates of *E. coli*, *K. pneumoniae*, *P. aeruginosa*, and *A. baumannii* infections were 75.5%, 73.9%, 89.5%, and 82.4%, respectively. For the 24 MDROs, the clinical response and treatment failure rates were 75.0% and 8.3%, respectively. Although the study group had a higher clinical response rate and lower treatment failure rate than the control group in the subgroup according to the causative pathogens, these differences were not statistically significant (Table 4).

### 2.5. Safety

Overall, the incidence of diarrhea, eosinophilia prolonged prothrombin time (PT), alteration in renal function and leukopenia was found in 23.2% (*n* = 36), 5.2% (*n* = 8), 3.2% (*n* = 5), 2.6% (*n* = 4), and 1.9% (*n* = 3) of all patients receiving cefoperazone-sulbactam, respectively. No significant difference was observed between the study and control groups in the risk of diarrhea (*p* = 0.326), eosinophilia (*p* = 1.000), prolonged PT (*p* = 0.674), alteration in renal function (*p* = 0.938) and leukopenia (*n* = 0.938) (Table 5). Finally, no gross bleeding was found in both the study and the control group

## 3. Discussion

This study compared the clinical efficacy and safety of two antibiotic strategies of cefoperazone-sulbactam in the treatment of acute bacterial infections in patients with CKD and had several significant findings. Most importantly, we found that the clinical efficacy of cefoperazone-sulbactam with a regimen of 2 g/2 g twice daily seemed to be better than those adjusted according to the renal functions, which was supported by the following pieces of evidence: First, patients receiving cefoperazone-sulbactam at 2 g/2 g twice daily had significantly higher clinical response rate and lower treatment failure rate than those who received a reduced dose of cefoperazone-sulbactam, according to their renal function. Second, although we found that the control group had more comorbidities and higher disease severity than the study group, the superior clinical efficacy in the study group remained unchanged after adjusting for disease severity and comorbidities. Third, although these kinds of differences were not statistically significant in the further subgroup analysis, the better outcome in the study group (without dose adjustment) than the control group (with reduced dose) remained consistent across all subgroups of different ages, sex, disease severity, and type of infections. Finally, a similar trend remained unchanged in the subgroup analysis according to the causative pathogens, including MDROs. All these findings could be explained by the hypothesis that a higher dose of cefoperazone-sulbactam could achieve a higher concentration at the infection sites and higher microbiological eradication. In summary, our findings indicated the better efficacy of cefoperazone-sulbactam at 2 g/2 g twice daily than at a reduced dose for treating an acute bacterial infection in CKD patients and suggested that there was no need to adjust the dose of cefoperazone-sulbactam in this clinical entity.

In addition to clinical efficacy, this study assessed the safety of unadjusted cefoperazone-sulbactam in patients with CKD. We found no significant difference in the risk of diarrhea, eosinophilia, prolonged PT, alteration in renal function, and leukopenia between the two strategies. In addition, there was no gross bleeding in both the study and the control group. This finding was consistent with previous clinical studies, which showed that the risk of AEs of cefoperazone-sulbactam was comparable to that of other antibiotics, such as cefepime and piperacillin-tazobactam [10,11,13,18,19,20]. However, this study is the first to demonstrate the tolerability of cefoperazone-sulbactam even without dose adjustment in patients with CKD and further suggested that cefoperazone-sulbactam at a dose of 2 g/2 g twice daily was tolerable for patients with CKD.

Our findings—Unadjusted cefoperazone-sulbactam may be more effective and not harmful for a patient with CKD than the adjusted dosage according to renal function could be explained by the following evidence. First, there is no need to adjust the dose of cefoperazone in patients with CKD. Second, the dose of sulbactam required adjustment according to the patient’s renal function, but a high dose of up to 6–9 g/day of sulbactam is needed to achieve a better response in the treatment of infection caused by MDROs [16,17].

This study has several limitations. First, the number of included patients was limited in this study; therefore, a significant difference was only observed in the overall population, but not in all subgroups analyses. Second, because the antibiotic susceptibility test was not routinely performed at the study site and the standard methods of measuring antibiotic susceptibility were lacking, we could not assess the effect of the antibiotic resistance pattern on the clinical outcome in this study. Further large-scale studies are required to clarify this issue.

## 4. Materials and Methods

### 4.1. Study Design

This study was conducted at Chi Mei Medical Center, a tertiary referral hospital with 1288 beds. Between 1 January 2015 and 30 July 2019, all patients who received cefoperazone-sulbactam for the treatment of acute bacterial infection were identified from the database of Chi Mei Medical Center. The inclusion criteria were as follows: (1) adult patients aged ≥20 years; (2) received cefoperazone-sulbactam for treating acute bacterial infection for at least 3 days; (3) the dose of cefoperazone-sulbactam was 2 g/2 g twice daily or adjusted according to renal function (1 g/1 g twice daily while creatinine clearance < 30 mL/min); and (4) creatinine clearance < 30 mL/min. The exclusion criteria were (1) patients with liver cirrhosis and (2) patients receiving anticoagulants. All data were collected on a routine basis, and the analysis was conducted retrospectively. This study was approved by the Institutional Review Board of Chi Mei Medical Center, and informed consent was waived (No. 10807-015).

### 4.2. Variable Measurement

We reviewed the medical records of all recruited patients and collected the following information: age, sex, type of infection, severity scores as SOFA score, underlying comorbidities or conditions, recent operation within three months, and Charlson score. Additionally, we collected data regarding causative pathogens, clinical response, risk of adverse events, and death on day 14.

### 4.3. Definitions

In this study, we defined patients receiving cefoperazone-sulbactam 2 g/2 g twice daily as the study group and those who received an adjusted dose of cefoperazone-sulbactam according to their renal function as the control group. We compared clinical outcomes (clinical response, treatment failure, and all-cause death) between the study and control groups. Clinical response was defined as the resolution or improvement of signs and symptoms of infection and no further antibiotic treatment after discontinuation of cefoperazone-sulbactam. In contrast, treatment failure was defined as clinical symptoms or signs that deteriorated or persisted during treatment and required additional antibiotics for management, death due to infection after 3 days of antibiotic treatment, or the development of complications. Mortality was defined as death from all causes.

### 4.4. Statistical Analysis

Continuous variables are presented as mean ± standard deviation, and categorical variables are reported as numbers (percentages). For univariate analysis, continuous data were compared using a *t*-test, and categorical data were compared using the χ^2^ or Fisher’s exact test, as appropriate. All univariate comparisons were unpaired, and all tests of significance were two-tailed. Logistic regression analysis was used to calculate the adjusted ORs and 95% CIs for the association between the dose of cefoperazone-sulbactam and patient outcomes after adjusting for age, sex, comorbidities, and disease severity. The linearity assumption between log odds of study outcomes and continuous independent variables, (e.g., age and body weight) was checked. Only variables in continuous scale that meet linearity assumptions were included in the multivariate logistic regression models.

## 5. Conclusions

Cefoperazone-sulbactam at a dose of 2 g/2 g twice daily could achieve better clinical efficacy than the reduced dosage regimen for the treatment of acute bacterial infection in patients with CKD. Additionally, this dosage did not increase the risk of AE compared to the reduced dose. Therefore, 2 g/2 g twice daily of cefoperazone-sulbactam is an effective and safe regimen for acute bacterial infection in patients with CKD.

## Figures and Tables

**Table 1:** The clinical characteristics of the patients.

Variables	Study Group *n* = 75	Control Group *n* = 80	*p*
Age, mean ± SD	78.5 ± 12.4	76.1 ± 12.0	0.226
Male sex, *n* (%)	37 (49.3%)	52 (65.0%)	0.049
Weight, mean ± SD	53.5 ± 11.4	58.2 ± 13.7	0.024
Type of infection, no (%)			0.004
Lower respiratory tract infection	23 (30.7%)	42 (52.5%)	
Urinary tract infection	36 (48.0%)	19 (23.8%)	
Others	16 (21.3%)	19 (23.8%)	
Comorbidities, no (%)			
Diabetes mellitus	28 (37.3%)	51 (63.8%)	0.001
Malignancy	24 (32.0%)	17 (21.3%)	0.129
Coronary artery disease	10 (13.3%)	17 (21.3%)	0.194
Heart failure	4 (5.3%)	18 (22.5%)	0.002
Chronic obstructive pulmonary disease	6 (8.0%)	11 (13.8%)	0.252
Liver cirrhosis	2 (2.7%)	3 (3.8%)	1.000 ^a^
End stage renal disease	0 (0.0%)	3 (3.8%)	0.246 ^a^
Rheumatological disease	0 (0.0%)	2 (2.5%)	0.497 ^a^
Alcoholism	1 (1.3%)	2 (2.5%)	1.000 ^a^
Recent surgery	4 (5.3%)	6 (7.5%)	0.747 ^a^
Charlson comorbidity index, mean ± SD	2.8 ± 2.0	4.1 ± 2.1	<0.001
SOFA score, mean ± SD	3.6 ± 2.1	5.0 ± 2.1	<0.001
eGFR	36.7 ± 17.2	21.6 ± 13.4	<0.001
Chronic kidney disease stage, *n* (%)			<0.001
Stage 4	60 (80.0%)	30 (37.5%)	
Stage 5 without dialysis	6 (8.0%)	24 (30.0%)	
Stage 5 with dialysis	9 (12.0%)	26 (32.5%)	
Antibiotic duration, mean ± SD	6.0 ± 2.4	7.9 ± 4.4	<0.001

^a^ Fisher’s exact test.

**Table 2:** Associations between the dosage of cefoperazone-sulbactam and clinical outcomes.

Outcomes	Study Group	Control Group	Crude OR (95% CI)	Adjusted OR (95% CI) ^a^
Clinical response	60 (80.0%)	52 (65.0%)	2.15 (1.04–4.46)	4.02 (1.49–10.81)
Treatment failure ^b^	3 (4.0%)	19 (23.8%)	0.14 (0.04–0.49)	0.06 (0.01–0.28)
Death	12 (16.0%)	9 (11.3%)	1.50 (0.59–3.80)	1.27 (0.35–4.64)

^a^ Adjusted for sex, weight, type of infection, SOFA score, diabetes mellitus, congestive heart failure, Charlson comorbidity index, the stage of chronic kidney disease, and antibiotic duration. ^b^ Excluded patients who died during follow-up period.

**Table 3:** Stratified analyses.

Variable	Clinical Response	Treatment Failure ^a^	Death
	OR (95% CI) ^b^	OR (95% CI) ^b^	OR (95% CI) ^b^
Sex			
Male	2.10 (0.73–6.05)	0.12 (0.01–0.99)	1.47 (0.39–5.49)
Female	2.79 (0.97–8.01)	0.12 (0.02–0.60)	1.36 (0.36–5.17)
SOFA score			
<4	1.79 (0.41–7.75)	0.15 (0.01–1.58)	2.13 (0.22–20.73)
≥4	1.91 (0.80–4.58)	0.16 (0.03–0.74)	1.67 (0.57–4.86)
Charlson comorbidity index			
≤3	2.51 (0.94–6.75)	0.05 (0.01–0.40)	3.10 (0.63–15.34)
>3	1.65 (0.51–5.33)	0.43 (0.08–2.23)	0.95 (0.22–4.11)
Chronic kidney disease stage, *n* (%)			
Stage 4	2.19 (0.85–5.63)	0.11 (0.02–0.59)	1.25 (0.40–3.95)
Stage 5 without dialysis	2.56 (0.28–23.7)	0.52 (0.05–5.00)	-
Dialysis			
No	1.71 (0.75–3.89)	0.14 (0.03–0.69)	1.49 (0.54–4.10)
Yes	5.87 (0.64–54.0)	0.21 (0.02–1.95)	-
Type of infection			
Lower respiratory tract infection	1.27 (0.41–3.96)	0.28 (0.03–2.57)	1.39 (0.39–5.00)
Urinary tract infection	1.62 (0.47–5.62)	0.16 (0.03–0.87)	-
Others	13.5 (1.47–123.7)	-	0.57 (0.05–6.90)

^a^ Excluded patients who died during follow-up period. ^b^ Crude odds ratios were presented.

**Table 4:** Subgroup analysis according to specific pathogens.

	All	Study Group	Control Group	*p*
***Escherichia coli*** **(*n* = 49)**				
Outcomes				0.132 ^a^
Clinical response	37 (75.5%)	24 (82.8%)	13 (65.0%)	
Treatment failure	6 (12.2%)	1 (3.5%)	5 (25.0%)	
Mortality	6 (12.2%)	4 (13.8%)	2 (10.0%)	
***Klebsiella pneumoniae*** **(*n* = 23)**				
Outcomes				0.366 ^a^
Clinical response	17 (73.9%)	11 (78.6%)	6 (66.7%)	
Treatment failure	2 (8.7%)	0 (0.0%)	2 (22.2%)	
Mortality	4 (17.4%)	3 (21.4%)	1 (11.1%)	
***Pseudomonas aeruginosa*** **(*n* = 19)**				
Outcomes				1.000 ^a^
Clinical response	17 (89.5%)	9 (90.0%)	8 (88.9%)	
Treatment failure	0 (0.0%)	0 (0.0%)	0 (0.0%)	
Mortality	2 (10.5%)	1 (10.0%)	1 (11.1%)	
***Acinetobacter baumannii*** **(*n* = 17)**				
Outcomes				1.000 ^a^
Clinical response	14 (82.4%)	4 (100.0%)	10 (76.9%)	
Treatment failure	1 (5.9%)	0 (0.0%)	1 (7.7%)	
Mortality	2 (11.8%)	0 (0.0%)	2 (15.4%)	
**MDROs ^b^ (*n* = 24)**				
Outcomes				0.226 ^a^
Clinical response	18 (75.0%)	7 (100.0%)	11 (64.7%)	
Treatment failure	2 (8.3%)	0 (0.0%)	2 (11.8%)	
Mortality	4 (16.7%)	0 (0.0%)	4 (23.5%)	

^a^ Fisher’s exact test. ^b^ Carbapenem-resistant *K. pneumoniae* (*n* = 5), carbapenem-resistant *E. coli* (*n* = 4), carbapenem-resistant *P. aeruginosa* (*n* = 2), carbapenem-resistant *E. cloacae* (*n* = 2), MDRO *A. baumannii* (*n* = 11).

**Table 5:** Overall summary of adverse events.

Adverse Event	Study Group *n* = 75	Control Group *n* = 80	*p*
Diarrhea	20 (26.7%)	16 (20.0%)	0.326
Eosinophilia	4 (5.3%)	4 (5.0%)	1.000 ^a^
Prolong PT	3 (4.0%)	2 (2.5%)	0.674 ^a^
Alteration in renal function	2 (2.7%)	2 (2.5%)	0.938 ^a^
Leukopenia	2 (2.7%)	1 (1.3%)	0.533 ^a^

^a^ Fisher’s exact test.
